# Complete Genome Sequences of Mycobacterium smegmatis Phages Chewbacca, Reptar3000, and Riparian, Isolated in Las Vegas, Nevada

**DOI:** 10.1128/MRA.01558-18

**Published:** 2019-02-07

**Authors:** Alicia Salisbury, Erin Cassin, Kevin Ayala-Pineda, Nicolas Barroga, Vanessa Cadiz, Ramiro Cisneros, Joseph Fersini, Tiffany Jeanite, Jonathan Juste, Juvie Ines, Gabriel Leyva, Dyanne Macalinao, Spencer Muscelli, Sophia Nhan, Gustavo S. Reyes, Heather Rhoden, Rodney Tan, Erika Torres, Krystal Tran, Georgette Uriarte-Valle, Christopher Wallace, Simon Wong, Kurt Regner, Christy Strong, Philippos K. Tsourkas

**Affiliations:** a School of Life Sciences, University of Nevada, Las Vegas, Nevada, USA; University of Southern California

## Abstract

Here, we present the complete genome sequences of Mycobacterium smegmatis phages Chewbacca, Reptar3000, and Riparian, isolated from soil in Las Vegas, NV. The phages were isolated and annotated by undergraduate students enrolled in the Phage Discovery course offered by the School of Life Sciences at the University of Nevada, Las Vegas.

## ANNOUNCEMENT

Of the roughly 2,500 sequenced phage genomes, approximately 1,600 belong to phages that infect Mycobacterium smegmatis (1). This is largely due to the versatility of this host and its consequent popularity with the Howard Hughes Medical Institute’s (HHMI) Science Education Alliance-Phages Hunters Advancing Genomics and Evolutionary Science (SEA-PHAGES) program (2). Here, we present the complete genomes of three Mycobacterium smegmatis phages isolated by students enrolled in the Phage Discovery course (BIOL 207X and BIOL 209X) at the University of Nevada, Las Vegas (UNLV).

Chewbacca and Reptar3000 were isolated from soil obtained from private homes in Las Vegas, while Riparian was isolated from soil collected near a pond in Las Vegas Wetlands Park. The phages were isolated, purified, and amplified in M. smegmatis MC^2^ 155 by students in the course BIOL 207X at UNLV using the protocols provided in HHMI’s SEA-PHAGES Phage Discovery Guide (https://seaphages.org/faculty/information/#phagediscovery). DNA was extracted using the manufacturer protocol provided in the Phage DNA isolation kit (catalog number 46800; Norgen Biotek). Phage genomes were sequenced at the University of Pittsburgh. Sequencing libraries were prepared from genomic DNA using a New England Biolabs (NEB) Ultra II kit producing 150-bp single-end reads. Libraries were sequenced with an Illumina MiSeq instrument, yielding single-end reads sufficient to provide at least 150-fold coverage for each genome. The reads were assembled using Newbler version 2.9 with default settings, in each case yielding a single contig which was checked for completeness, accuracy, and phage genomic termini using Consed version 29 as described in Russell (3).

The assembly results and the phages’ GenBank and SRA accession numbers are shown in Table 1. Phages were assigned to clusters based on genomic sequence similarity using the PhagesDB.org database and the Phamerator software with default settings (1, 4). Despite their similar geographic provenances, the phages are not closely related. Chewbacca is a member of cluster N; Reptar3000, cluster K4; and Riparian, cluster R. A ClustalW multiple alignment using default settings showed that all three phage pairs have roughly 40% average nucleotide sequence identity (ANI) between them, which is the same as that of two randomly generated DNA sequences. Chewbacca and Reptar3000 use the “cohesive ends with 3′ overhangs” DNA packaging strategy, while Riparian uses the “circularly permuted ends” strategy (5).

**TABLE 1:** Phage GenBank and SRA accession numbers and genome assembly results

Phage name	GenBank accession no.	SRA accession no.	Avg coverage (×)	Cluster	Genome length (bp)	GC content (%)	No. of genes
Chewbacca	MH926055	SRX4721842	3,156	R	43,575	66.2	74
Reptar3000	MH926058	SRX4721843	946	K4	54,601	67.6	89
Riparian	MH926059	SRX4721841	1,158	N	71,199	56.0	101

The assembled genomes were annotated using DNA Master with default settings, as described in Pope and Jacobs-Sera (6), by students in the course BIOL 209X at UNLV. We identified 74 genes in Chewbacca, 89 in Reptar3000, and 101 in Riparian. Protein functions were assigned using BLAST, CD-Search, and HHPred. Using a cutoff *E* value of 1E−3, we assigned putative function to about half of the genes in Chewbacca and Reptar3000 and to a third of the genes in Riparian. A small and a large terminase, portal protein, major capsid protein, several head-tail connector complex proteins, major tail protein, two tail assembly chaperones, tail tape measure protein, several minor tail proteins, and a lysin A were identified in all three phages. None of these exhibit sequence conservation across the three phages as determined by ClustalW alignment using default settings (<25% amino acid sequence identity). A lysin B was identified in Reptar300 and Riparian, a holin and an integrase in Chewbacca and Reptar3000, a Cro in Reptar 3000, an excise in Chewbacca and Reptar3000, and a tRNA-Lys(ttt) in Reptar3000. The tail assembly chaperones have a predicted translational frameshift located in the 3′ region of the upstream tail assembly protein.

### Data availability.

GenBank and SRA accession numbers are listed in Table 1.
